# A Bio-Inspired Approach for the Reduction of Left Ventricular Workload

**DOI:** 10.1371/journal.pone.0087122

**Published:** 2014-01-24

**Authors:** Niema M. Pahlevan, Morteza Gharib

**Affiliations:** 1 Medical Engineering, Division of Engineering & Applied Sciences, California Institute of Technology, Pasadena, California, United States of America; 2 Graduate Aerospace Laboratories, Division of Engineering & Applied Sciences, California Institute of Technology, Pasadena, California, United States of America; University of Perugia, Italy

## Abstract

Previous studies have demonstrated the existence of optimization criteria in the design and development of mammalians cardiovascular systems. Similarities in mammalian arterial wave reflection suggest there are certain design criteria for the optimization of arterial wave dynamics. Inspired by these natural optimization criteria, we investigated the feasibility of optimizing the aortic waves by modifying wave reflection sites. A hydraulic model that has physical and dynamical properties similar to a human aorta and left ventricle was used for a series of in-vitro experiments. The results indicate that placing an artificial reflection site (a ring) at a specific location along the aorta may create a constructive wave dynamic that could reduce LV pulsatile workload. This simple bio-inspired approach may have important implications for the future of treatment strategies for diseased aorta.

## Introduction

Congestive Heart Failure (CHF) is a condition in which the heart fails to circulate enough blood in the vascular networks. CHF has reached an epidemic level where the number of patients suffering from this condition in the U.S. alone is more than five million and growing [1]. Clinical investigations have confirmed that pulsatile load plays an important role in the pathogenesis of left ventricular hypertrophy (LVH) and the progression of LVH to CHF [2], [3], [4]. The pulsatile load is the result of the complex dynamics of wave propagation and reflection in compliant arterial vasculature [5], [6]. Significant efforts have been made to understand the wave dynamics of the arterial system and to clarify its role in heart failure and other cardiovascular diseases [2], [3], [4], [7], [8], [9].

The pulsatile load on the left ventricle is controlled by the wave dynamics of the arterial vasculature [5], [6]. Wave dynamics in a compliant tube is mainly dominated by three parameters: (1) fundamental frequency (or wavelength) of the waves; (2) wave speed (which is defined by material properties of the tube); and (3) the location of reflection sites [10], [11], [12]. Similarly, wave dynamics in the aorta and arterial network is determined by heart rate (HR), pulse wave velocity (PWV), and reflection sites. The interplay among these three parameters defines a wave dynamics condition where the pulsatile workload on the heart is minimized. Using a simplified computational model of the aorta we have previously shown the interplay among these wave dynamic parameters results in an optimum HR in which the pulsatile workload is minimized [13]. There were several limitations involved with this computational study; therefore, it is necessary to confirm the finding using a physiologically relevant experimental model. Our main objective in this manuscript is to introduce a bio-inspired approach to reduce the pulsatile workload. In this manuscript, we also present validation of the finding of our previous computational study (the optimum HR concept) using an in-vitro experimental approach.

Previous studies have demonstrated the existence of optimization criteria in design and development of mammalians cardiovascular system [14], [15], [16], [17], [18], [19]. Arterial wave dynamic parameters such as the reflection coefficient [20], normalized input impedance[21], pulse wave velocity[17], [18]; as well as the product of the propagation constant and the aortic length [20] are all invariant of mammalian size. These similarities in mammalian arterial wave reflection suggest there are certain design criteria for the optimization of arterial wave dynamics. Quick *et al* [22] showed that wave reflections are optimized in animals under normal physiological conditions. They also showed that either reducing or increasing wave reflections results in an elevation of LV pulsatile workload [22]. Their study suggests that the mammalian arterial system is designed to optimize the wave reflections rather than minimize them. Inspired by this natural optimization criterion, we will investigate in this study if it is possible to optimize the aortic waves by simply modifying reflection sites in order to reduce the pulsatile workload on the heart.

## Materials and Methods

### Equipment and Materials

**Figure 1:**
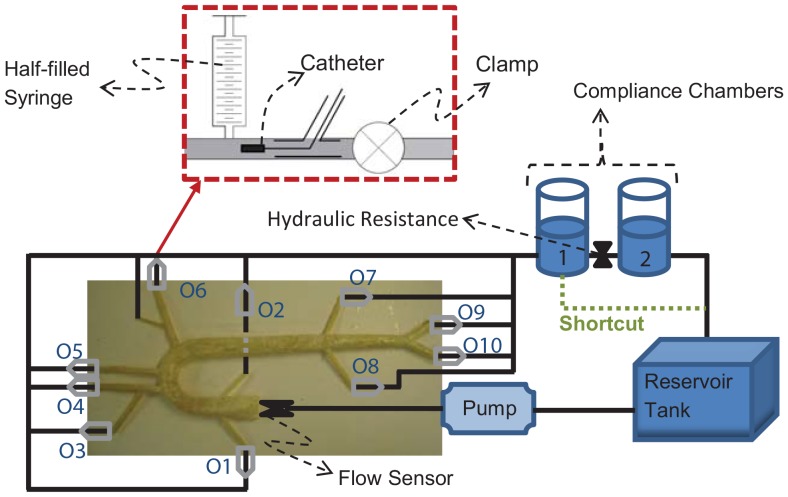
Schematic of the Aortic Simulator. Schematics of the outlet units are shown in the dashed-red box. The numbers at the outlets correspond to the value given in Table 3.

An experimental hydraulic model called the aortic simulator was used in this study (Figure 1). The aortic simulator is a hydraulic model that has physical and dynamical properties similar to a human aorta and left ventricle which can be used for the *in-vitro* hemodynamic studies.

**Figure 2:**
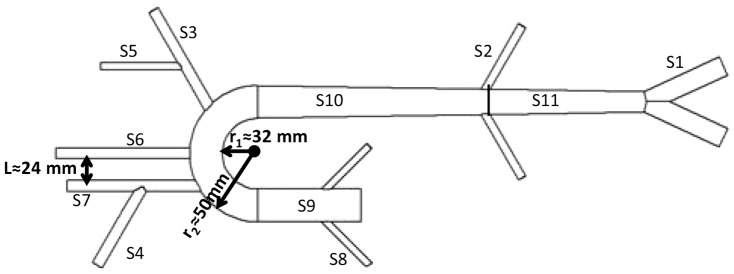
Schematic of the aortic mold. Dimensions are provided in Table 1.

The left ventricle was simulated by a piston-in-cylinder pump (ViVitro Labs Inc. SuperPump System: Model SPS3891) that generates the pulsatile flow (using a programmed waveform generator WG5891) and sends it into a compliant aorta. The artificial aortas were built based on a 1-1 scale of a human aorta mold and it includes major branches of the aorta, aortic arch, and aortic tapering. Figure 2 and Table 1 provides the schematic and the dimensions of the aortic mold which was used in this study to create the aortas. Different compliant models of the artificial aorta were made from clear natural latex (Chemionics Corp.) and silicone (39 Shore A Hardness RTV Silicone). We made aortas with different compliances by changing the number of applications of dipping (for latex aortas) or coating (for silicone aortas). Different compliances resulted in different wave speeds or pulse wave velocities (PWV). Relevant dynamical and physiological characteristics of these aortas such as characteristic impedance (*Z_c_*), aortic compliance (*AC*), and PWV are provided in Table 2. The foot-to-foot method [23] was applied to compute the PWV of each aorta (Table 2). The aortic rigidities (PWV) used in this study belong to healthy humans older than 60. However, under disease conditions such as hypertension and diabetes, the aortic rigidity increases considerably [24].

**Table 1:** Geometrical properties of the aortic mold.

Segment No.	Artery	Length	Diameter
*S1*	Iliac	*76 (mm)*	*12 (mm)*
*S2*	Renal	*65 (mm)*	*5 (mm)*
*S3*	Left subclavian	*100 (mm)*	*7 (mm)*
*S4*	Right subclavian	*65 (mm)*	*7 (mm)*
*S5*	Vertebral	*65 (mm)*	*5 (mm)*
*S6*	Left carotid	*100 (mm)*	*7 (mm)*
*S7*	Right carotid	*100 (mm)*	*7 (mm)*
*S8*	Coronary	*45 (mm)*	*4 (mm)*
*S9*	Ascending aorta	*70 (mm)*	*24 (mm)*
*S10*	Thoracic aorta	*170 (mm)*	*20* (start) *–24* (end) *(mm)*
*S11*	Abdominal aorta	*130 (mm)*	*16* (start) *–20* (end) *(mm)*

**Table 2:** Dynamical and physiological properties of the artificial aortas.

Aorta No.	*Z_c_ (dyne.sec.cm^−3^)*	*AC* (*cm^5^.dyne^−1^*)	*PWV* (*m.s^−1^*)	material
M1	670	64.5×10^−5^	6.7	*Silicone*
M2	860	54.7×10^−5^	8.6	*Latex*
M3	950	32.2×10^−5^	9.5	*Latex*
M4	1140	49.5×10^−5^	11.4	*Silicone*
M5	1300	30.7×10^−5^	13	*Latex*
M6	1350	30.7×10^−5^	13.5	*Latex*
M7	1500	23.2×10^−5^	15	*Latex*
^a^Physiological range	(500–1500)	(0.34–2.35)	(5–24)	

*Zc* is characteristic impedance, *AC* is aortic compliance, and *PWV* is pulse wave velocity. Normal Physiological ranges are taken from O'Rourke and Hashimoto[30], Liu *et al.* [31], Murgo *et al*. [32], and Safar and O'Rourke [24]. ^a^These ranges are different under disease conditions.

A unit was designed to the end of each outlet that mimics the resistance and compliance of the eliminated vasculature. This unit includes one syringe, one clamp, and one port for catheter insertion (Figure 1). The syringe is half-filled with air which provides the required compliance of the eliminated vascular network (see Table 2). The added end-compliance depends on the mean pressure and air volume in the syringes (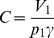, see the Supporting Information file for the derivation). The compliance values are provided in Table 3. The clamp was to increase the terminal resistance. The aortic simulator also includes two compliance chambers with hydraulic resistance in between that were installed at the end of the aortic loop. These chambers enabled us to control the total volume compliance (the values are provides in Table 3). The reservoir tank is the last component of the aortic simulator. It connects the second chambers to the inlet of the pump (Figure 1).

**Table 3:** Volume compliance by air column.

Outlet location	air volume (*mL*)	*VC_1_* (*cm^5^.dyne^−1^*)	*VC_2_* (*cm^5^.dyne^−1^*)
Location O1 and O2	*6*	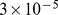	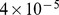
Location O3 and O6	12	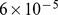	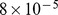
Location O4 and O5	15	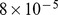	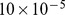
Location O7 and O8	15	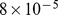	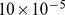
Location O9 and O10	24	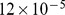	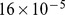
Cylindrical Chamber 1	1402	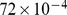	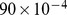
Cylindrical Chamber 2	1402	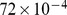	N.A^a^
Total Volume Compliance		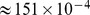	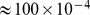

*VC_1_* is the volume compliance at the mean pressure of *p_mean_*≈104.5 mmHg (setup1; see Measurement and Procedures section). *VC_2_* is the volume compliance at the mean pressure of *p_mean_*≈83 mmHg (setup1; see Measurements and Procedures). For outlet location See Figure 1. ^a^Cylindrical Chamber 2 was removed in setup 2 (see Measurements and Procedures)

### Measurements and Procedures

The pressure data was measured at the aortic input and outlet of the compliant aorta (outlet 9 in Figure 1) using Swan-Ganz catheters (Swan-Ganz 116F4 pediatric double lumen monitoring catheter at the inlet and Swan-Ganz 116F5 pediatric double lumen monitoring catheter at the outlet, Edwards Life Sciences) and Utah Medical Disposable pressure transducers (DPT-400). The signals were collected using the Tri-pack pressure measuring systems (TP8891, Vivitro Labs Inc.). The Tri-pack system consists of three bridge amplifiers. H16XL Transonic flow sensor (Transonic Systems Inc.) in combination with a T110 Transonic Bypass flow meter was used to measure the volume flow rate at the inlet. The pressure and flow measurements have been done simultaneously. The pressure and flow data were collected for 4 seconds and each experiment was repeated at least 5 times.

Experiments were completed at various heart rates (ranging from 60 bpm to 200 bpm) with seven different aortas (see Table 2). In all experiments, water was used as the circulating fluid and any visible air bubbles were removed prior to the experiments. The pump was operating under 40% systole (waveform C, waveform generator WG5891) in all experiments. Cardiac output (CO) was kept constant (≈*5 L/min*) in all experiments. We used three experimental setups in this study. These setups are as follows:

Setup 1: This setup is the same as the one shown in Figure 1. This setup included two compliance chambers and the resistance between the two.

Setup 2 (low volume compliance and low resistance setup): In this setup, the first chamber was shortcut to the tank (green dashed-line in Figure 1). Since the second chamber and the resistance between chambers were removed in this setup, the aortic simulator had lower total volume compliance and lower resistance compared to setup 1 (the mean pressure in setup 1 and setup 2 were 104.5±3.5 mmHg and 84±2 mmHg respectively).

**Figure 3:**
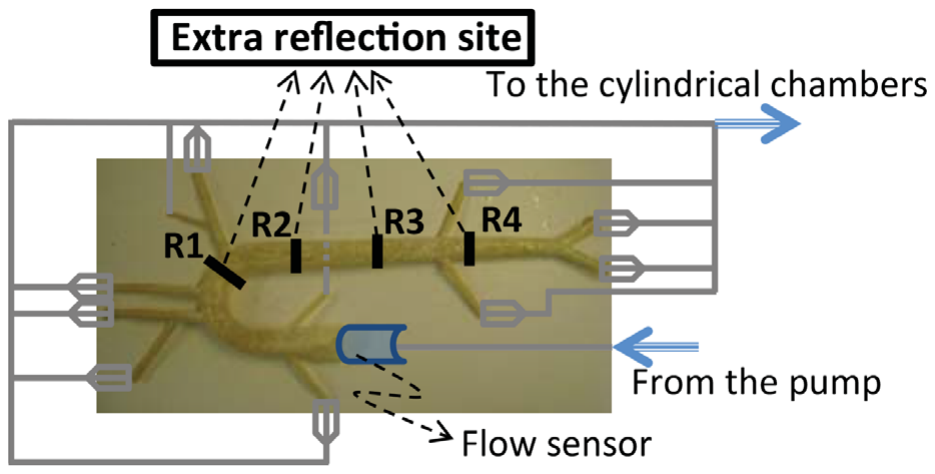
The aortic simulator for setup 3. An extra reflection site (a ring) was placed at different locations along the aorta marked by numbers 1–4. They are located at approximately 15, 25, 35 and 45 cm from the aortic input.

Setup 3: This is the setup for the reflection site experiment. It is similar to setup 1. In this setup, an extra reflection site (a ring) was placed at different locations along the aorta as illustrated in Figure 3 (The ring was just a snap grip hose and it was located outside of the aorta). This extra reflection site was used to alter the aortic wave reflection. The pressure and flow data were collected for 4 seconds and each experiment was repeated 9 times.

#### Power calculation

The pulsatile power was calculated using equation (1):
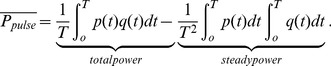
(1)


Here, *T* is the period of the cardiac cycle, *p(t)* is the pressure, and *q(t)* is the flow. The pressure and flow were measured at the aortic input.

#### Pulsatile power-wave reflection relation

The pulsatile power can also be written as a summation of the pressure and flow harmonics[18] as
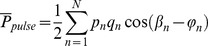
(2)where 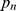
and 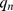
are the harmonic the pressure and flow wave, respectively, and 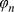 and 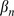
are the phases of the pressure and flow, respectively.

The reflection coefficient (*R*) is defined as the ratio of the harmonics of the reflected pressure (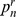) to the harmonics of the forward pressure (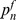) in the frequency domain as [25]

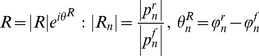
(3)


It can also be written in terms of the impedances as [25]

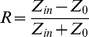
(4)


Where 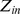 is the input impedance, 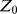
is the characteristic impedance, and “
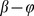
” is the phase of the input impedance (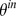) of the system. Therefore,
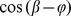
can be computed as
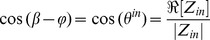
(5)where 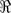 is the symbol denoting the real part of a complex function. Solving equation (4) for 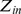 gives
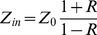
(6)when combined with equation (4) and (5), it gives
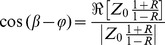
(7)


In the case of an aorta where viscoelastic properties are negligible, the characteristic impedance is real (it has a negligible imaginary part) [22]. Hence, in this condition, equation (7) can be simplified as
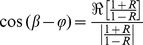
(8)


Using the impedance definition, the ratio of flow and pressure are related as
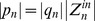
(9)


Finally, substituting equation (8) and equation (9) into equation (2), noting the relations 
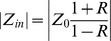
and 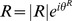, results in
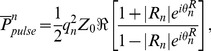
(10)where, for simplicity, we have shown only one harmonic of the series representation for pulsatile power.

To find the condition where an increase in reflection will result in a decrease of the input pulsatile power, we assumed a flow source condition for the heart (*q_n_* is constant) and considered the function (for simplicity, 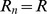 and 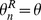):
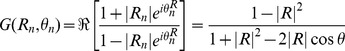
(11)


## Results

### Physiological Relevancy of the Aortic Simulator

**Figure 4:**
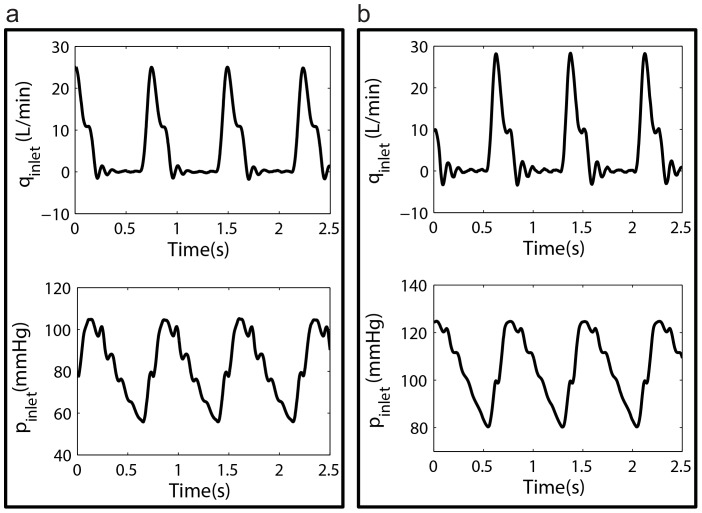
Samples of hemodynamic waveforms. a) A sample of an aortic input flow wave (top) and a sample of the aortic input pressure wave (bottom) at *HR = 72 bpm* and *CO = 5 L/min* for setup 2 b). A sample of an aortic input flow wave (top) and a sample of the aortic input pressure wave (bottom) at *HR = 80 bpm* and *CO = 5 L/min* for setup 1.

**Figure 5:**
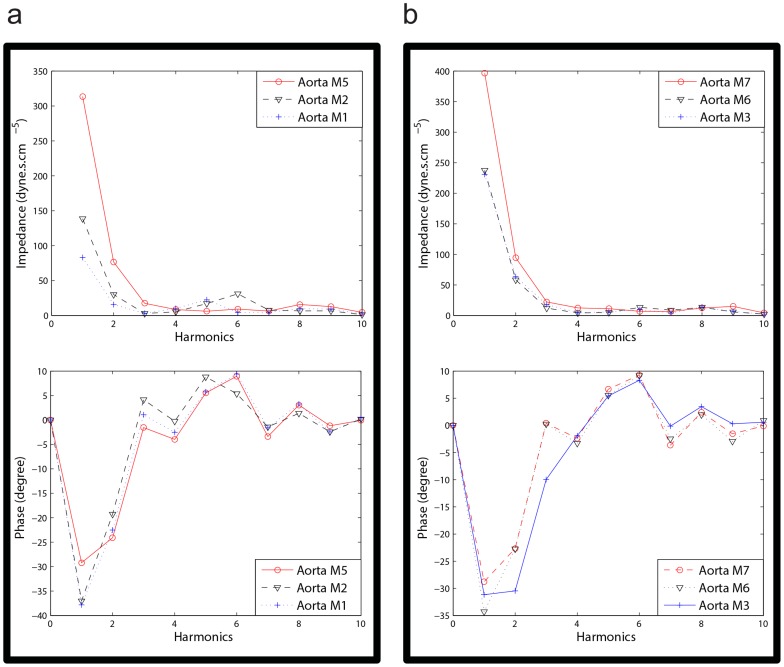
Aortic input impedance. a) Sample impedance modulus (top) and phase (bottom) of three aortas in setup2 computed at *CO = 5 L/min* and *HR = 72 bpm*. b) Sample impedance modulus (top) and phase (bottom) of three aortas in setup1 computed at *CO = 5L/min* and *HR = 72 bpm*.

Sample measured flow and pressure waveforms from setup 2 and setup 1 are provided in Figure 4a and 4b respectively. The modulus and phase of the input impedance of the aortas under both setup2 and setup 1 are also shown in Figure 5a and Figure 5b respectively. Relevant hemodynamical parameters such as pulse pressure (*PP*), mean pressure (*P_mean_*), and total compliance (*TC* = *AC*+ total *VC*) for all aortas are provided in Table 4. Total resistance (*TR*) for setup1 (*TR* = *Δp*/(Cardiac output)) is 1518.3 (*dyne sec cm^−5^*). For setup 2 the *TR* is 1174.3 (*dyne sec cm^−5^*). These *TR* values are both within physiological range, 700<TR<1600 (*dyne sec cm^−5^*) [26].

**Table 4:** Hemodynamic properties of the aortic simulator.

Aorta No.	*TC_1_*	*TC_2_*	*PP_1_*	*PP_2_*	*P_mean1_*	*P_mean2_*
M1	NA	1065×10^−5^	NA	22.0	NA	82.4
M2	NA	1055×10^−5^	NA	40.0	NA	81.9
M3	1543×10^−5^	1033×10^−5^	44.3	49.3	104.3	81.0
M4	1559×10^−5^	1049×10^−5^	53.9	55.7	102.4	85.1
M5	1541×10^−5^	1031×10^−5^	61.3	75.2	108.7	85.5
M6	1541×10^−5^	1031×10^−5^	63.3	75.9	109.1	85.5
M7	1533×10^−5^	1023×10^−5^	94.6	106	101.4	82.9

*TC_1_* (*cm^5^.dyne^−1^*), *PP_1_* (*mmHg*), and *P_mean1_* (*mmHg*), are for setup1. *TC_2_* (*cm^5^.dyne^−1^*), *PP_2_* (*mmHg*), and *P_mean2_* (*mmHg*), are for setup2.

### Effect of Heart Rate and Aortic Rigidity on Left Ventricular Pulsatile Workload

**Figure 6:**
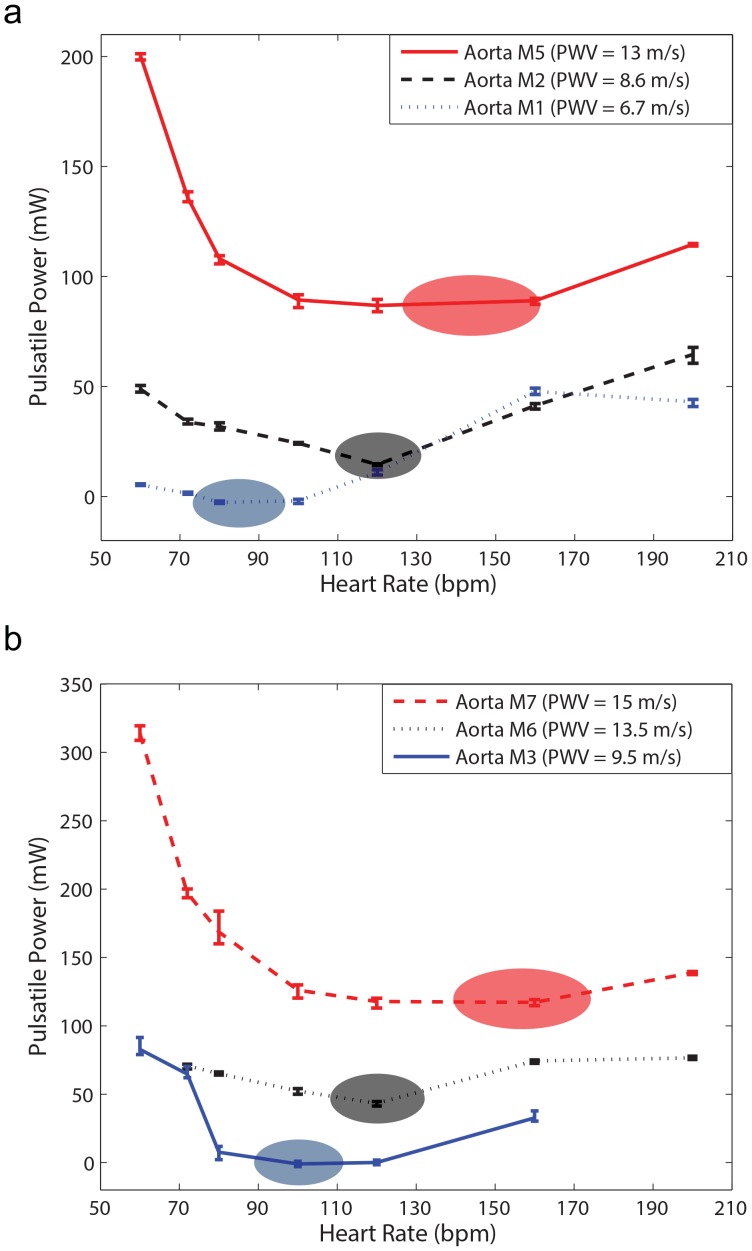
Pulsatile power versus heart rate (HR). a) Results of Setup2 (low TC condition) for three different aortic rigidities; *CO = 5 L/min* for all data points (please see supplementary data file for all other aortas). b) Results of Setup1 (high TC condition) for three different aortic rigidities; *CO = 5 L/min* for all data points (please see supplementary data file for all other aortas). There is an optimum HR in which pulsatile power is minimized. As the aortic rigidity increases, the optimum HR shifts to a higher value. Pulse wave velocity (*PWV*) is the wave speed and it is an index for aortic rigidity. Each power data point is the result of the respective experiment repeated five times.

Figure 6 demonstrates the effect of aortic rigidity and heart rate (HR) on input pulsatile power. Figure 6a shows the results of setup 2 that have lower total volume compliance and higher resistance compared to setup 1 (Figure 6b). These figures show that there is an optimum HR at each level of aortic rigidity in which pulsatile external power (pulsatile workload) is minimized. The optimum HR has a higher value in more rigid aortas (Figure 6a and 6b). These results are in agreement with the results of our previous computational study[13].

### Effect of Total Volume Compliance and Resistance on Optimum HR

**Figure 7:**
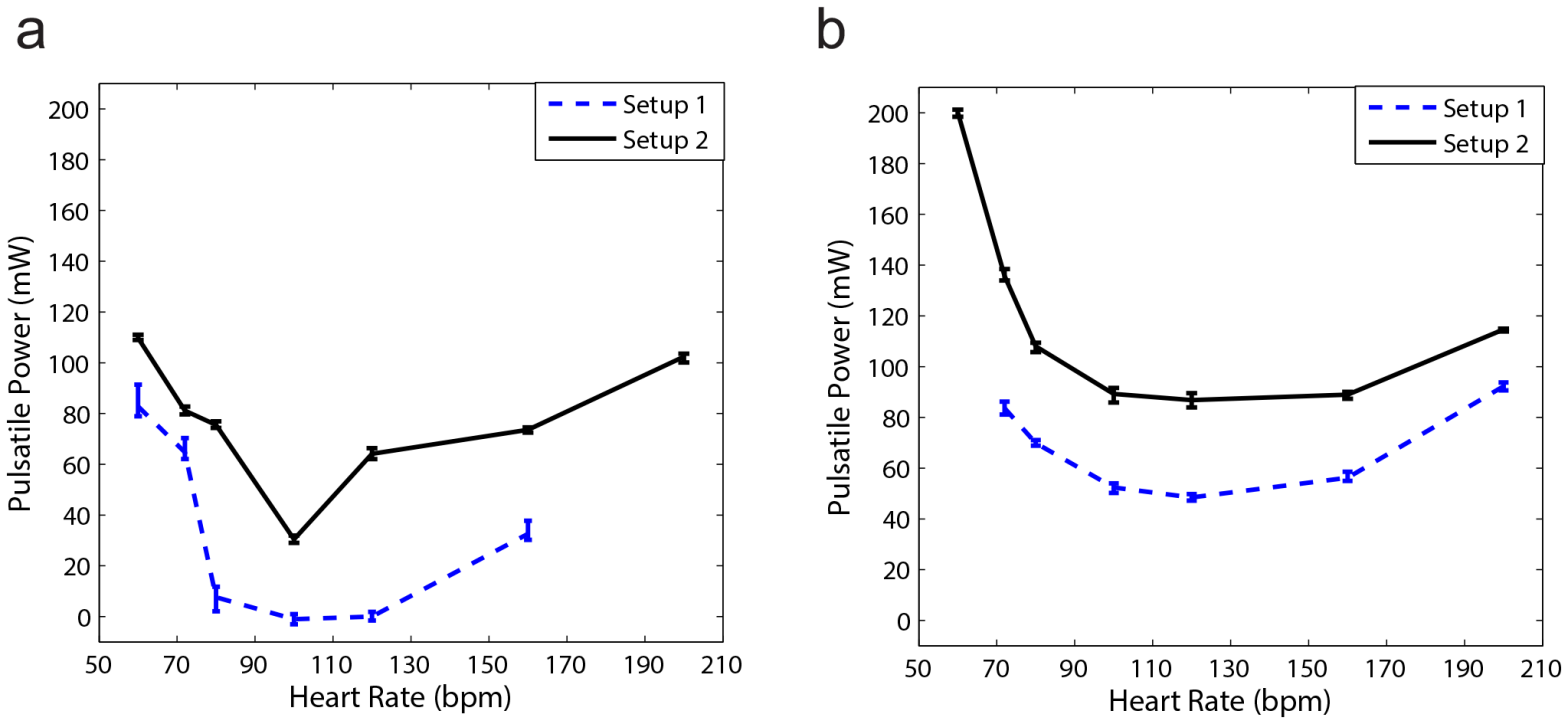
The effect of total volume compliance and total resistance on optimum *HR*. a) Pulsatile power versus *HR* for the aorta M3 with *PWV = 9.5 m/s*. b) Pulsatile power versus *HR* for the aorta M 5 with *PWV = 13 m/s*. Changing total resistance and total compliance affect mean, pulse pressure, and pulsatile power; however, they do not alter the optimum *HR* (optimum wave condition). Each power data point is the result of the respective experiment repeated five times.

Figure 7a and 7b show the effect of total volume compliance and peripheral resistance on the optimum value of *HR* for two aortas with different rigidities (M3 with *PWV = 9.5 m/s* and M5 with *PWV = 13 m/s*). Although changing total resistance and total compliance alters mean and pulse pressure (as can be seen in Figure 4 and Table 4), the optimum wave condition (that results in optimum *HR*) does not depend on total volume compliance and total resistance as demonstrated in Figure 7. Due to the limitations of our pump, we performed the experiments at particular discrete heart rates. As a result, the exact optimum *HR* sometimes could not be identified.

### Bio-inspired approach: Optimizing the Location for Reflection Sites

**Figure 8:**
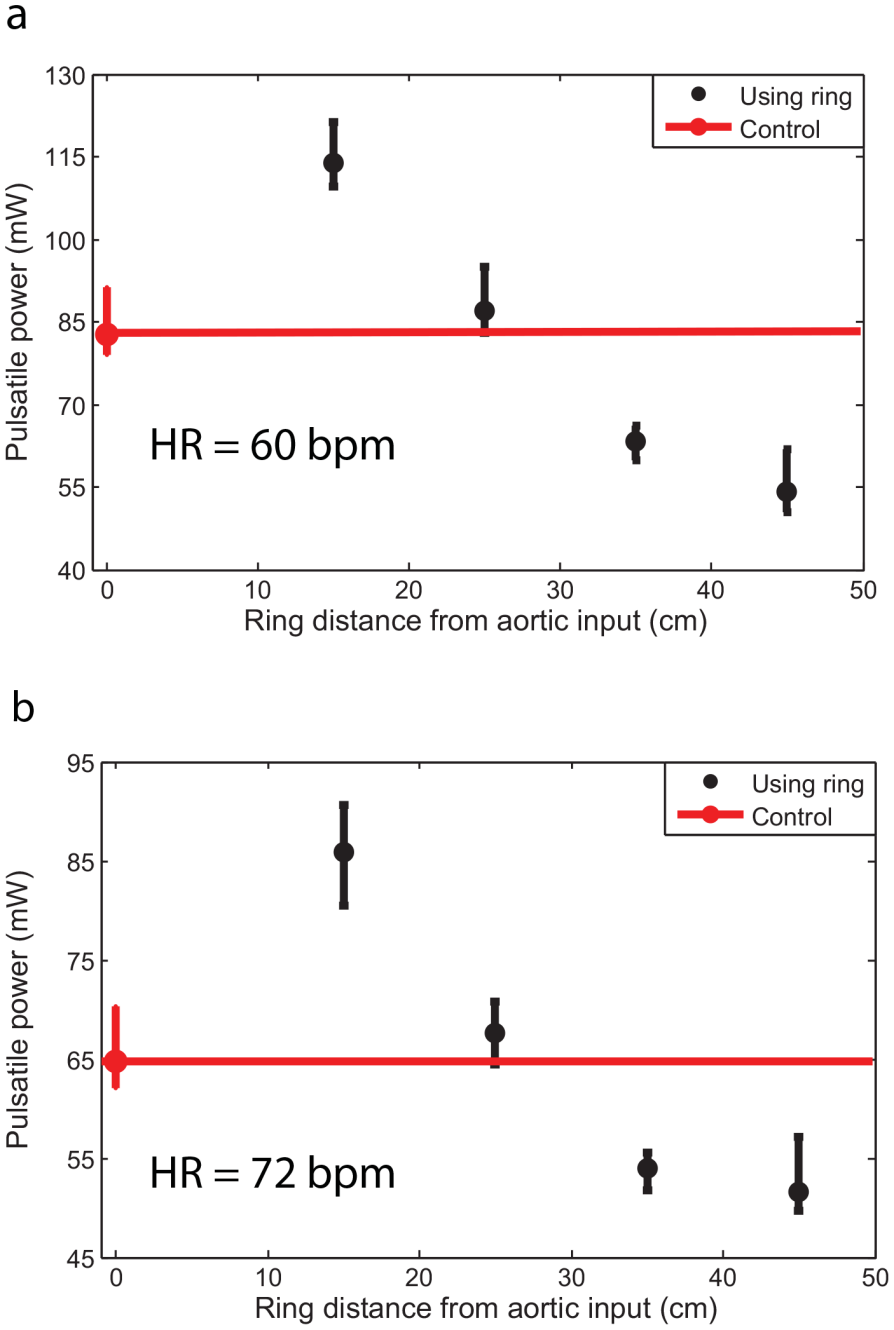
The effect of an extra reflection site created by a ring on input pulsatile power (see Materials and Methods section). (a) Results are for Aorta 3 (see Table 2) with *PWV = 9.5 m/s* and *HR = 60 bpm*. (b) Results are for Aorta 3 with *PWV = 9.5 m/s* and *HR = 72 bpm*. The control case is the aorta without an extra reflection site and the red line is the pulsatile power of the aorta without any rings (i.e. no extra reflection sites). The pulsatile power can increase or decrease compared to the control case (control is the aorta without the ring), the nature of which depends on the location of the ring. Each data point is the result of the respective experiment repeated nine times.

**Figure 9:**
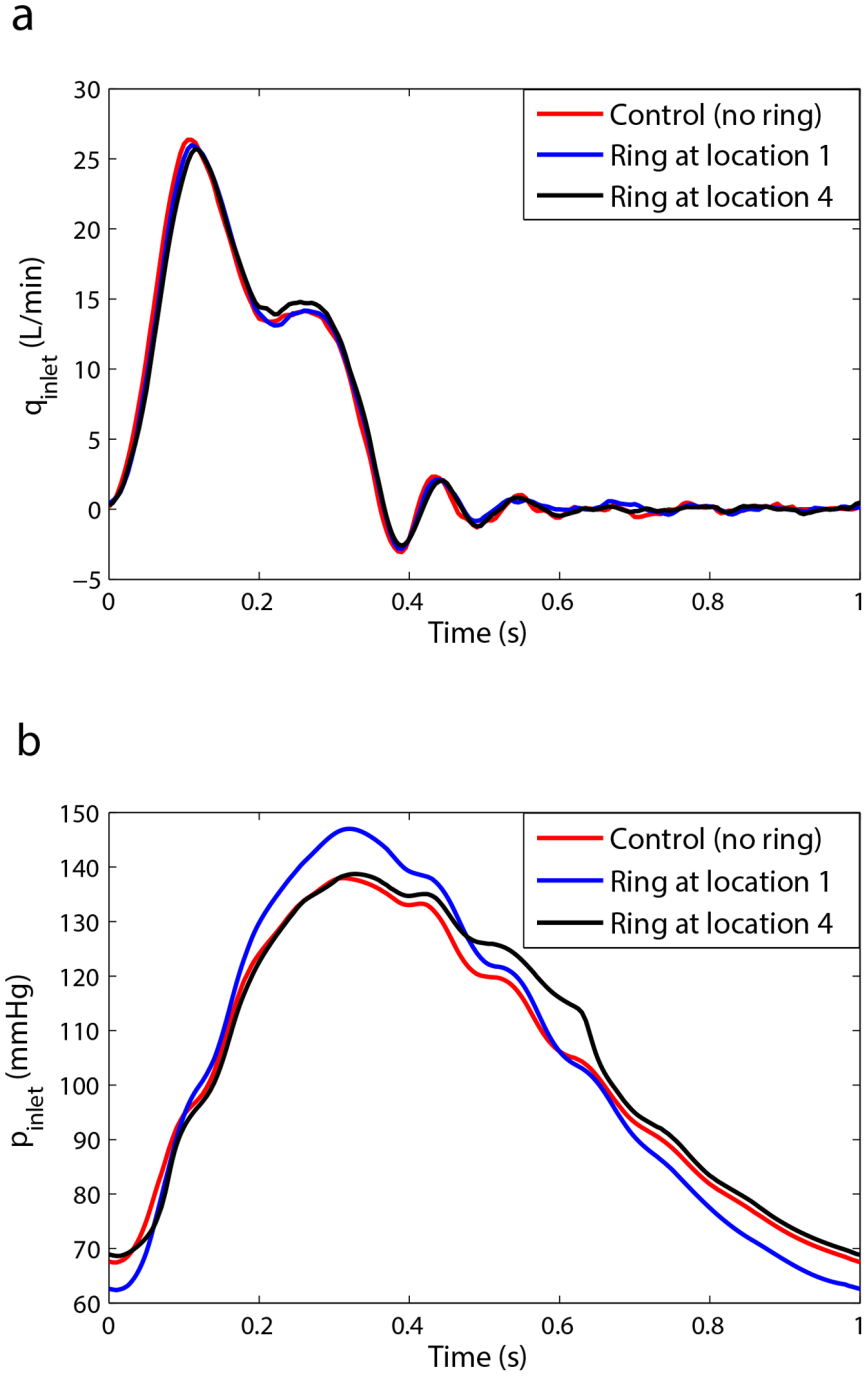
Samples of hemodynamic waveforms for ring experiment. a) Samples of aortic input flow waves at *HR = 60 bpm* and *CO = 5 L/min* for setup 3 (ring experiment). b) Samples of aortic input pressure waves at *HR = 60 bpm* and *CO = 5 L/min* for setup 3 (ring experiment). Since our LV simulator acts more as a flow source, the alterations from control case are insignificant in flow waves. However, the alterations from the control case in pressure waves are rather significant.

As shown in previous sections, a specific combination of the three wave parameters (HR, PWV, and location of reflection site) creates a condition in which the LV pulsatile power is minimized. In this section, we tested the hypothesis that the reflection sites can be modified to improve the effect of wave reflection. Figure 8 demonstrates that placing an extra reflection site (a ring) at a particular location along the aorta could reduce the LV pulsatile power (workload). This has been shown for two different HRs in Figure 8(a) and 8(b) where it is clear that the pulsatile power can increase (destructive wave dynamics) or decrease (constructive wave dynamics) based on the location of the new reflection site. Samples of measured flow and pressure waveforms (with and without the ring) at the aortic input location are provided in Figure 9a and 9b respectively (please see supplementary data file for all other cases). It can be observed that the changes in the flow waveforms are insignificant since our LV simulator (which is a piston-type pump) acts more like a flow source. However, the changes in the pressure wave forms are noteworthy as shown in Figure 9b.

**Figure 10:**
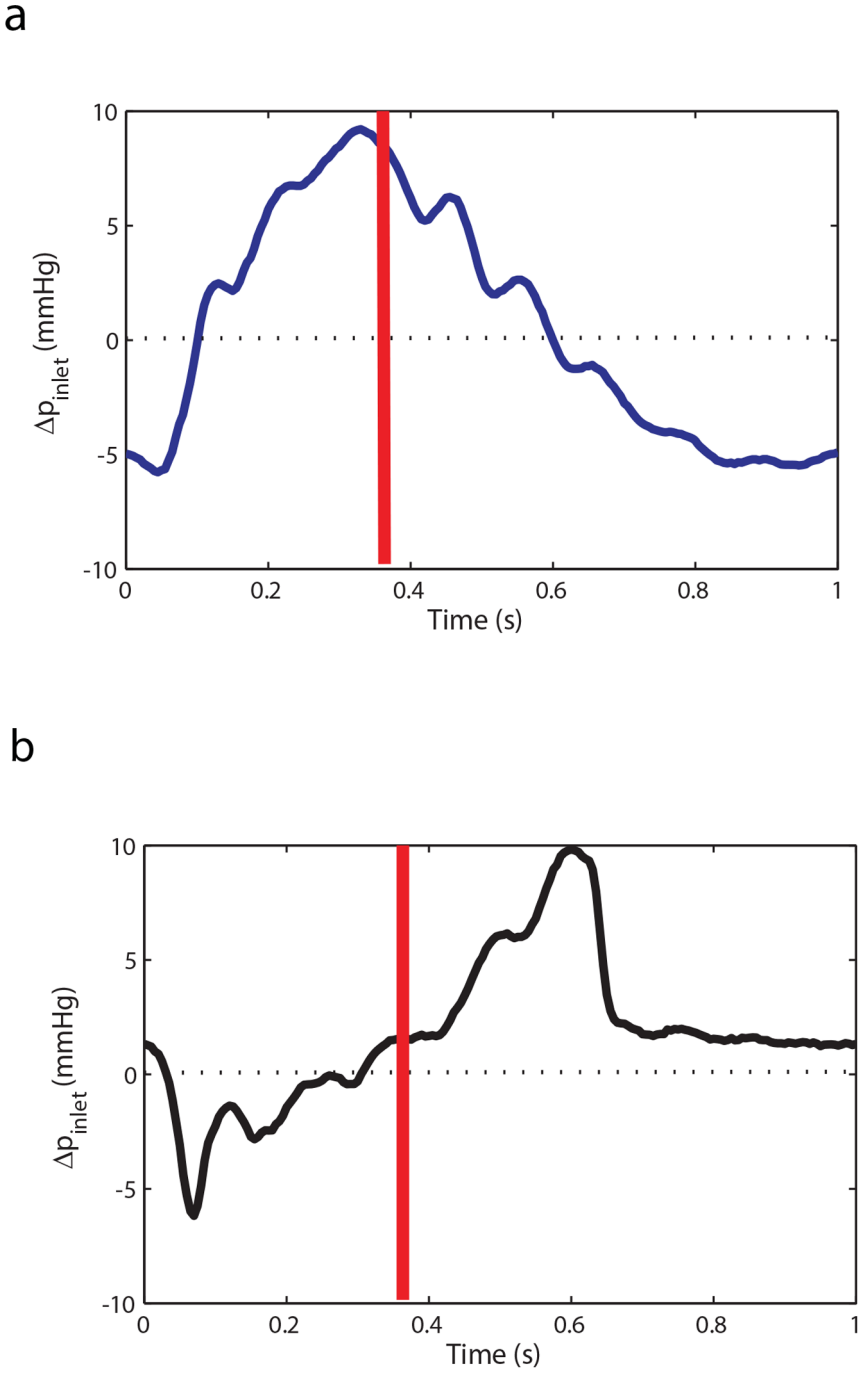
Pressure wave difference for ring experiment. a) The input pressure wave difference of ring 1 (destructive case) from the control (Δ*p_ring1_ = p_ring1_−p_control_*) at *HR = 60 bpm* and *CO = 5 L/min* for a complete cardiac cycle. b) The input pressure wave difference of ring 4 (constructive case) from the control (Δ*p_ring4_ = p_ring4_−p_control_*) at *HR = 60 bpm* and *CO = 5 L/min* for a complete cardiac cycle.

Figure 10 shows the pressure wave difference (Δ*p = p_ring_ −p_control_*) throughout a cardiac cycle at the aortic input. As shown in Figure 10 (a), when the ring is located at position 1, the pressure wave difference (Δ*p_ring1_ = p_ring1_−p_control_*) is mainly positive during the systolic phase, where the end of the systolic phase is marked by the red line. This excess systolic pressure increases the left ventricle pulsatile power. In contrast, when the ring is located at position 4, the pressure wave difference (Δ*p_ring4_ = p_ring4_−p_control_*) is mostly negative during the systolic phase of the cycle and as a result the left ventricle pulsatile power decreases. Interestingly, when the ring is at location 4, the diastolic pressure wave difference (excess pressure during diastole) is positive as shown in Figure 10 (b). Although this excess diastolic pressure may change in an *in-vivo* physiological system the presence of this excess pressure in the in-vitro experiment suggests that in certain cases the ring may also prove to be beneficial by increasing the perfusion of blood to the coronary arteries.

#### Pulsatile power and wave reflection

According to equation (11), the region where 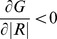 corresponds to the range of 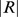 and 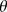
where pulsatile power is decreasing while wave reflection is increasing. This region depends on both the magnitude of the wave reflection (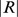) and its phase (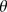), and is described as
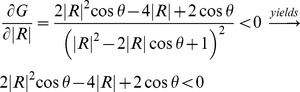



**Figure 11:**
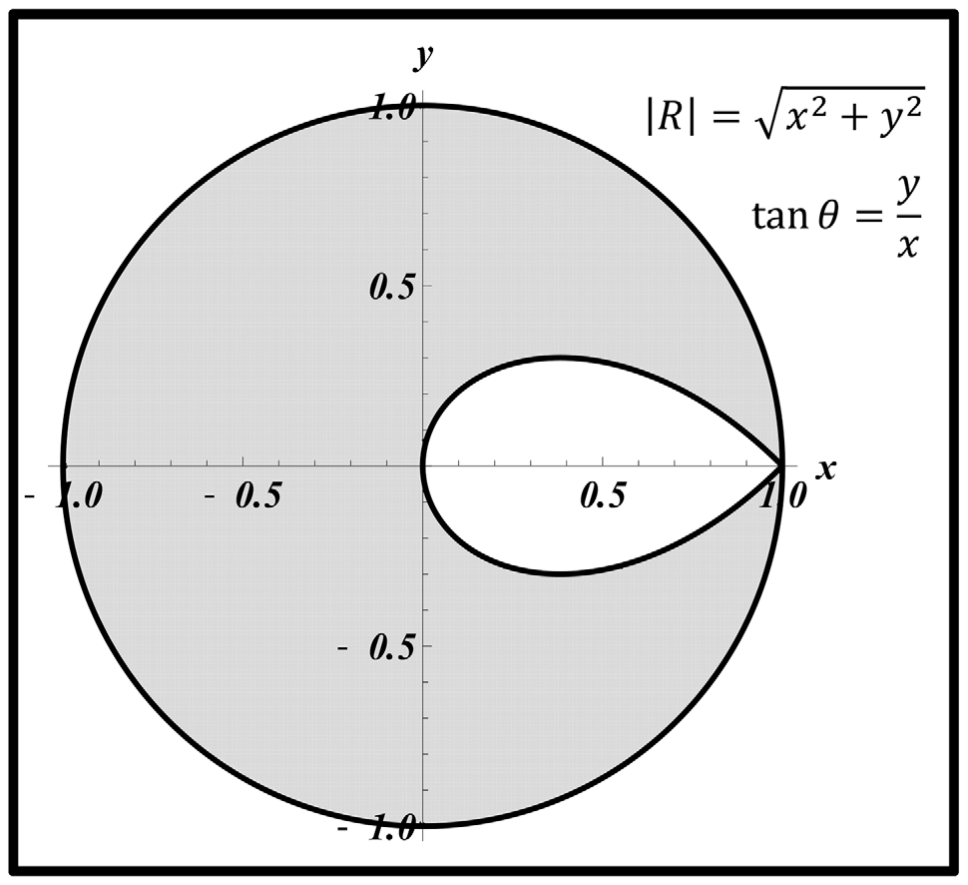
The grey area is the region where increasing wave reflection is beneficial. In this region, increasing wave reflection results in a decrease of the input pulsatile power (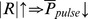). In the white region, increasing wave reflection is disadvantageous and results in an elevation of the pulsatile power (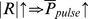).

Obviously for any value of 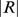, this condition is satisfied if 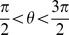 since 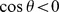 and 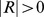. This means that increasing the wave reflection causes a decrease in pulsatile power when the phase falls in the second or third quadrant. In the first and fourth quadrants (where 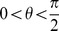 and 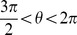 respectively), increasing the wave reflection can increase or decrease (depending on the magnitude and phase) the input pulsatile power. This is illustrated in Figure 11, the grey area denoting the range of 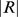
and 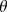 where pulsatile power decreases by the act of increasing the wave reflection. Similar analyses have been done by Quick *et al* [22]. However, our contour for the boundary between the two regions has a teardrop shape, whereas the one presented by Quick *et. al.* is circular [22] (we have found it necessary to show the details of our derivation in the method section since our final graph is different from the one presented by Quick *et al*; however, they didn’t provide the details).

## Discussion

### Optimum Heart Rate for Left Ventricle Pulsatile Workload

Using an experimental approach, we have shown in Figure 6a and 6b that there is an optimum HR at each stage of aortic rigidity in which the LV external pulsatile power (LV pulsatile workload) is minimized. This optimum HR shifts to higher values as aortic rigidity increases. It can be concluded that the interplay between HR and wave speed (which depends on the wall rigidity) changes the optimum point. These findings confirm earlier results that were obtained from our computational study [13].

The wave dynamics in a compliant tube is mainly dominated by the frequency of excitation (HR), the wave speed, and the reflection sites as shown in previous studies [10], [11], [12]. Similarly, aortic wave dynamics is controlled by the heart rate (HR), the pulse wave velocity (PWV), and the locations of the reflection sites. The optimum aortic wave condition depends on these interrelated parameters. Figure 7a and 7b show that parameters such as total volume compliance and total resistance do not affect the optimum HR value (However, they do affect the workload on the heart). In fact, the value of the optimum HR only depends on wave dynamic parameters such as PWV (wave speed) as demonstrated in Figure 6a and 6b. As expected, the pulsatile power-HR curves shift up when total compliance decreases (Figure 7a and 7b).

### A Bio-inspired Approach: Correction and Optimization of Aortic Waves

Traditionally, there was a misconception that wave reflections have only negative effects and that reducing the wave reflections is always beneficial. However, Zamir [27] proposed that wave reflections can in fact be beneficial and can even assist blood flow rather than impeding it. Not long after, Quick *et al* [22] showed that either increasing or decreasing wave reflection results in the elevation of pulsatile workload. Based on this observation, they have concluded that arterial wave dynamic is in optimum condition under normal physiological conditions. This inspired us to investigate if it is possible to reduce LV pulsatile workload through the correction and optimization of aortic wave dynamics. Under healthy condition, this workload accounts only for 6–12% of the total LV workload [28]. However, the pulsatile workload significantly increases under vascular disease conditions [24].

Optimization of wave reflections as a therapeutic approach was first suggested by O'Rourke [29]. We have shown in this manuscript that the introduction of properly positioned extra reflection sites in the aorta can result in a constructive wave dynamic state and a subsequent reduction of LV pulsatile workload. To test this idea, a ring (rigid reflection site) was placed at various locations along the aorta to alter the dynamics of wave reflection. Decreasing the pulsatile load in a heart failure patient is critically important [2], [3]. HF is usually accompanied by increased arterial stiffness. It is clinically impractical to increase the HR in order to reach a new optimum HR. Therefore our proposed “reflection site modification” method can potentially be used for reduction of the LV pulsatile workload for HF patients.

Figure 8a and 8b show that alteration of the wave reflection site can result in either an increase (a destructive effect) or decrease (a constructive effect) of the LV pulsatile power. The constructive and destructive effects of waves depend on the location of the ring (reflection site) since different locations cause different wave interactions. In other words, the phase of the global reflection coefficient varies with the location of the extra reflection site. To understand this phenomenon, we looked into the relation between pulsatile power and wave reflection. Our analysis depicted in Figure 11 can be used to explain the observed phenomena in Figure 8(a) and 8(b). In our experiments, when the ring was located at Position 1 (15 cm from the input, see Figure 3) the operation point of the system was in a state found in the white area (Figure 11); hence, increasing the wave reflection increased the pulsatile power. At Position 2 (25 cm from the input), the system is found to be in the boundary between the two regions of Figure 11 where the pulsatile power does not change significantly with the increased reflection. For Positions 3 and 4, the system lies in the operational region similar to the gray area where increasing wave reflection results in a decrease of pulsatile power. Under this beneficial wave condition, an extra reflection site reduces the input pulsatile power as shown in Figure 8(a) and 8(b).

### Limitation

The major limitation of our study is related to the fact that an in-vitro model of the systemic arterial system was considered. In fabricating the synthetic aortas used in this study the focus was on mimicking the material-dependent parameters such as the average aortic pulse wave velocity and volume compliance. In this regard, a synthetic aorta fabricated by dip molding and composed of homogenous material properties may not be able to exactly mimic the local variations in PWV and compliance present in an in-vivo system although the average PWV and volume compliances may match. Physiologically, the human aorta consists of multiple segments with variable material compositions which would result in specific pulse wave velocity variations at different segments along its length. Therefore, the dynamics of the LV-arterial system in a true physiological situation are likely to be different from our *in-vitro* experimental model. This means that the optimum HR and optimum ring location in our model may not be exactly the same as the *in-vivo* situation.

Another limitation of our study is related to oversimplification of microvasculature in our model; however, this simplification does not change the main finding of this study since microvasculature does not influence the aortic wave dynamics. The microvascular network only contributes as a discrete reflection site and as a resistance to blood flow, and both of these effects are properly modeled in our in-vitro experiment. We also used water as a circulatory fluid in this study, but this does not affect our results since the fluid viscosity plays a negligible role in the dynamics of aortic waves[5]. Our model includes a piston pump. This assumes that heart is a flow source. The heart is neither a flow nor a pressure source, but the behavior of a normal heart is closer to a flow source [4]. Our system is not an exact duplicate of an in-vivo model, but the physiological relevancy of our aortic simulator has been shown (Tables 2–4 and Figures 3–4).

## Conclusion

Using an in-vitro experimental approach we have validated what we proposed in our previous computational study [13]. We showed in this manuscript that there is an optimum heart rate at which the pulsatile workload on the left ventricle is minimized. The optimum heart rate shifts to a higher value as the aortic rigidity increases.

A simple bio-inspired concept, based on the principles of wave dynamics, was also introduced to reduce the LV workload in heart failure patients. A device based on this concept could be in the form of a ring or a band wrapped around the aorta to act as an extra reflection site that alters wave reflection. This device can be designed to be minimally invasive due to its lack of complexity. However, the effectiveness of such a device is yet to be determined and is the subject of future work.

## Supporting Information

Appendix S1 (DOC)

Data S1 (ZIP)
