# Supplementary material for: A Bio-Inspired Approach for the Reduction of Left Ventricular Workload
Source: PLoS One. 2014 Jan 24;9(1):e87122. doi: 10.1371/journal.pone.0087122 (PMC3901771; doi:10.1371/journal.pone.0087122)
Supplement: Appendix S1 — (DOC) [file pone.0087122.s001.doc]

*Volume compliance produced by air column*

According to the gas law:

$pV^{\gamma}=K$, (S1)

where *p* is the pressure *V* is the volume, $\gamma$ is the adiabatic index (=1.2-1.4 for air) and *K* is a constant. Volume compliance (*C*) can be calculated form volume (*V*) and pressure (*p*) as:

$C=\left|\frac{dV}{dp}\right|$, (S2)

By combining equation (S1) and (S2), one can derive:

$C=\frac{\left(\frac{k}{p}\right)^{1/\gamma}}{p\gamma}$. (S3)

An approximate formula around a certain mean pressure, *p_1_*, and mean volume, *V_1_*, can be derived as

$C=\frac{V_{1}}{p_{1}\gamma}$. (S4)
